# Ocular trauma among patients attending a tertiary teaching hospital in Zimbabwe

**DOI:** 10.1371/journal.pone.0292392

**Published:** 2023-10-04

**Authors:** Samuel Kyei, Michael Agyemang Kwarteng, Frederick Afum Asare, Moses Jemitara, Claudio Ngoni Mtuwa

**Affiliations:** 1 Department of Optometry, Faculty of Science and Engineering, Bindura University of Science Education, Mashonaland Province, Zimbabwe; 2 Department of Optometry and Vision Science, College of Allied Sciences, University of Cape Coast, Cape Coast, Ghana; 3 Biomedical and Clinical Research Centre, College of Health and Allied Sciences, University of Cape Coast, Cape Coast, Ghana; 4 The Eye Department, Bongo District Hospital, Bongo, Upper East Region, Ghana; LV Prasad Eye Institute, INDIA

## Abstract

**Purpose:**

To investigate the trends, prevalence and distribution of ocular trauma in a Zimbabwean Tertiary Teaching Hospital (Parirenyatwa).

**Method:**

A hospital-based retrospective cross-sectional study was conducted at the Parirenyatwa Group of Hospitals, Sekuru Kaguvi Eye Department in Harare, Zimbabwe, to review medical records of patients with ocular trauma visiting for treatment at the outpatient department between January 2017 and December 2021. Information on patients’ demographics, presenting visual acuity, type of ocular trauma, and the number of eyes affected were collected and analysed using descriptive and inferential statistics.

**Results:**

A total of 863 patients (1007 eyes) were identified to have experienced ocular trauma of one kind or another, with the youth (18–35 years) reporting with most cases (331, 38.4%). About 71.2% of patients were classified as having open-globe injuries and of that number, 90% were caused by blunt trauma, while the rest were caused by penetrating, intraocular, and perforating injuries. Patients with open-globe injuries were about 10 times more likely to develop blindness than those with closed-globe injuries after adjusting for age and gender, and this was statistically significant (ARR = 9.65, 95% CI: 5.53–16.84, p < 0.001). The prevalence of distance vision impairment due to ocular trauma was 60.1% (95% CI: 56.8%-63.4%), with majority resulting in blindness (22.0%, 95% CI: 19.4%-24.9%).

**Conclusion:**

There is a high prevalence of open-globe injuries in Zimbabwe with blunt trauma being the most significant cause. This suggests the need to promote and intensify public eye health awareness and sensitisation on safety strategies for the prevention of ocular trauma throughout the country.

## Introduction

Ocular trauma is a public health concern and a significant cause of vision impairment globally, even though most cases are usually preventable [1, 2]. The primary cause of unilateral vision loss, particularly in developing nations, is trauma [3]. These injuries contribute significantly to blindness globally, with substantial financial ramifications for patients and society [4]. According to Thylefors [5], ocular trauma accounts for 5% of all ophthalmology hospitalisations in developed nations, while it contributes to about one-third of presenting cases of severe visual impairment in developing nations.

An injury involving deep layers of the cornea and other parts of the globe can cause permanent disability, including blindness, reduced vision, and other complex eye conditions that medication and spectacles cannot correct [6]. Head injury, on the other hand, may result in traumatic optic neuropathy, which, if untreated with steroids or surgery, could lead to blindness [7]. Importantly, traumatic visual loss generally depends on the projectile’s impact [8].

It has been found that every year, more than 55 million people are reported to experience ocular trauma globally, including about 200,000 cases of open-globe injuries and 750,000 of these cases necessitating hospitalisation [9]. Surprisingly, most of these cases have been reported to be more common in developing countries like Zimbabwe [10]. The only study conducted in Zimbabwe on ocular injuries is more than two decades ago [11] thus, the need to investigate and report the current trend. This study provides epidemiological data on the trends, prevalence and distribution of eye trauma utilising data from a tertiary hospital’s database. By this, many ocular injuries, mostly preventable and treatable, could be tackled to alleviate the permanent vision loss they might cause.

## Materials and methods

The study was a hospital-based retrospective cross-sectional study at the Parirenyatwa Group of Hospitals, Sekuru Kaguvi Eye Department. Parirenyatwa is a major referral hospital situated in the capital city, where many activities exist. A convenient purposive sampling method was used in collecting the required data. This was done to ensure only ocular trauma cases that reported to the hospital within the stipulated time were included in the study.

### Inclusion and exclusion criteria

Patients’ records that were seen from January 2017 to December 2021 were included in the study, including full medical records of all patients with ocular injuries in the hospital’s register. Hospital registers without complete information on ocular trauma (47) including the type of injury, affected eye, among others were, however, excluded. Data collection spanned from August 2022 through November 2022.

### Data collection process

Patients’ information, including demographics (age, gender, year of visit), presenting visual acuity (monocular), cause of trauma, type of ocular trauma, and the number of eyes affected, were collected using a well-designed datasheet by one of the researchers (JM). This was then double-checked by a second researcher (MAK). All medical records were in hard copies as the hospital did not use an electronic medical record system. Data were then input in a Microsoft spreadsheet which was further exported to STATA 16.1 (StataCorp LLC, College Station, TX, USA) for analysis.

Ocular injury was then classified under two subgroups (open- and closed-globe injuries) using a classification system employed by Dogramaci, Erdur and Senturk [12]. Open-globe injuries were defined as eye damage with full-thickness injuries in the eye wall (corneoscleral), while closed-globe injuries were defined as wounds without complete thickness in the eye wall. However, four categories of open-globe injuries were created based on the mechanism of injury. Type A injuries were full-thickness wounds from a blunt object (rupture). Type B was assigned to single and complete thickness injuries brought on by sharp objects in the eye wall (penetrating injury). Type C injuries included those involving an object in the eye (intraocular foreign body). Double full-thickness eye wall injuries that served as both an entry and an exit site were categorised as type D injuries (perforating injury) [12].

### Ethical consideration

Ethical approval (approval ID: 0020/2022) for the study was obtained from the Institutional Review Board of the Bindura University of Science Education, while permission was obtained from the Parirenyatwa Group of Hospitals, Sekuru Kaguvi Eye Department. To maintain anonymity and confidentiality, each record card was assigned a unique identification number, and participants’ records were not exposed or given out at any point in the data collection process. Authors also had no access to information that could identify participants during or after data collection.

### Data analysis

Data were analysed with STATA 16.1 (StataCorp LLC, College Station, TX, USA). Descriptive and inferential statistics were conducted, and mean, standard deviations, percentages and proportions were reported. Prevalence and 95% confidence intervals were also computed, while multinomial regression analyses were conducted to assess the relationship between various categorical variables. Normality of data was assessed with the Shapiro-Wilk test while statistical significance was set at p < 0.05.

## Results

### Baseline characteristics of patients

A total of 33,694 patients visited the eye hospital between January 2017 and December 2021, with 910 patients having recorded histories of ocular injuries and 863 having complete information for the study. Of the 863 patients included in the study, 580 (67.2%) were males, while the rest were females. The mean ± standard deviation (SD) age of all patients attended to at both hospitals was 29.3 ± 17.7 years, with the ages ranging from day 0 to 87 years. Although, on average, males were slightly older (29.7 ± 17.4 years) than females (28.7 ± 18.1 years), this was not statistically significant (mean diff: 1.0 ± 1.3 years, t = 0.81, df = 861, p = 0.42). Patients were classified into children (0–17 years), youth (18–35 years), adult (36–59 years) and elderly (60 years and above) and these are presented in Table 1. Out of 1,007 eyes affected by ocular trauma, a little over 70% affected either the right or left eye (monocularly), while the rest affected both eyes (binocularly). This is also described in Table 1.

**Table 1:** Distribution of ocular trauma according to age groups, gender and eye affected.

**Age groups (N = 863)**	**Male N (%)**	**Female N (%)**	**Total N (%)**
Children (0–17 years)	160 (18.5)	81 (9.4)	241 (27.9)
Youth (18–35 years)	213 (24.7)	118 (13.7)	331 (38.4)
Adult (36–59 years)	174 (20.2)	64 (7.4)	238 (27.6)
Elderly (60+ years)	33 (3.8)	20 (2.3)	53 (6.1)
Total	580 (67.2)	283 (32.8)	863 (100.0)
**Affected eyes (N = 1007)**			
Right Eye	225 (22.3)	116 (11.5)	341 (33.9)
Left Eye	264 (26.2)	114 (11.3)	378 (37.5)
Both Eyes	182 (18.1)	106 (10.5)	288 (28.6)
Total	671 (66.6)	336 (33.4)	1007 (100.0)

**Fig 1:**
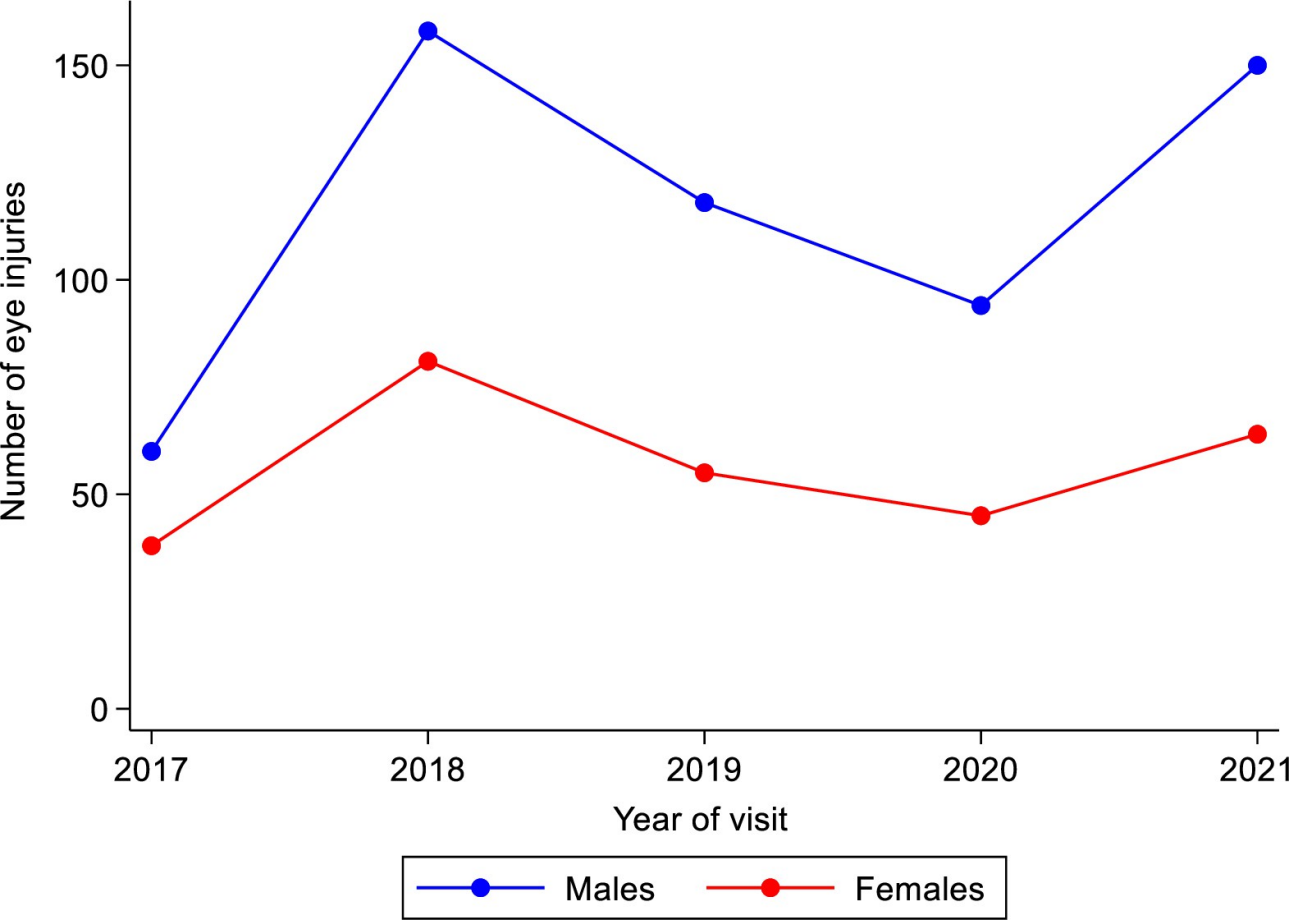
Distribution of reported cases of ocular trauma during each reviewed year by gender.

A majority (239, 27.7%) of eye injuries were reported in 2018, with the least (98, 11.4%) reported in 2017. For each year, it was found that there were more males with ocular trauma than females; however, there was no specific trend in the recorded cases of ocular trauma for the years under review (2017–2021). Among the reported causes of ocular trauma were injuries from assault, chemicals (including acid), fall, fishhook, foreign body, and fight. Fig 1 illustrates the distribution of reported cases of ocular trauma during each reviewed year by gender.

### Classification of ocular trauma

Of all the patients with ocular trauma, 71.2% were classified as having open-globe injuries (eye damage with full-thickness injuries in the eye wall (corneoscleral)), while the rest were classified as having closed-globe injuries (wounds without complete thickness in the eye wall). Among those with open-globe injuries, rupture/blunt trauma was found to be the most common cause accounting for about 90%, while penetrating injury, intraocular injury and perforating injury accounted for approximately one-tenth. There were more males with both open (48.7%) and closed-globe injuries (18.5%) than females (22.5%—open-globe injuries; 10.3% -closed-globe injuries), but this was not statistically significant (X^2^ = 1.38, df = 1, p = 0.24). Table 2 describes the classification and distribution of patients with ocular trauma. There was a statistically significant difference between open- and closed-globe injuries across the different age groups (X^2^ = 13.5, df = 3, p = 0.004).

**Table 2:** Classification and distribution of ocular trauma by gender.

**Types of ocular trauma (N = 863)**	**Male N (%)**	**Female N (%)**	**Total N (%)**
Open-globe injury	420 (48.7)	194 (22.5)	614 (71.2)
Closed-globe injury	160 (18.5)	89 (10.3)	249 (28.8)
Total	580 (67.2)	283 (32.8)	863 (100.0)
**Causes of open-globe injury (N = 614)**			
Rupture/blunt injury (Type A)	374 (60.9)	176 (28.7)	550 (89.6)
Penetrating injury (Type B)	18 (2.9)	10 (1.6)	28 (4.6)
Intraocular injury (Type C)	17 (2.8)	4 (0.65)	21 (3.4)
Perforating injury (Type D)	11 (1.8)	4 (0.65)	15 (2.4)
Total	420 (68.4)	194 (31.6)	614 (100.0)

### Prevalence of presenting vision impairment due to ocular trauma

Patients’ presenting distance visual acuities due to ocular trauma were used to categorise them into the various types of distance vision impairment as described by the World Health Organisation (International Classification of Diseases 11 [2018]). From the analysis, the prevalence of presenting vision impairment resulting from ocular trauma was 60.1% (95% CI: 56.8%-63.4%). While the most prevalent vision impairment was blindness 22.0% (95% CI: 19.4%-24.9%), the least was severe vision impairment 2.8% (95% CI: 1.9%-4.1%). Table 3 shows the distribution and prevalence of the different types of presenting vision impairment.

**Table 3:** Distribution and prevalence of presenting vision impairment due to ocular trauma.

Presenting VI	Frequency (N = 863)	Prevalence	95% CI
Normal	344	39.9	36.6–43.2
Mild VI	119	13.8	11.6–16.3
Moderate VI	186	21.6	18.9–24.4
Severe VI	24	2.8	1.9–4.1
Blindness	190	22.0	19.4–24.9

VI: Vision impairment, VA: visual acuity, N: Number of patients, CI: Confidence Interval, Normal: Snellen VA better than or equal to 6/12, Mild VI: Snellen VA worse than 6/12 to 6/18, Moderate VI: Snellen VA worse than 6/18 to 6/60, Severe VI: Snellen VA worse than 6/60 to 3/60, Blindness: Snellen VA worse than 3/60.

### Relationship between type of ocular trauma and vision impairment

A chi-square test revealed that there was a statistically significant difference between vision impairment patterns between patients with open-globe injuries and closed-globe injuries (X^2^ = 100.98, df = 4, p < 0.001) (Table 4). Multinomial regression analysis further revealed that compared to patients with closed-globe injury, those with open-globe injury were 3 times more likely to develop mild vision impairment (ARR = 2.92, 95% CI: 1.81–4.69, p < 0.001), 4 times more likely to develop both moderate (ARR = 3.55, 95% CI: 2.31–5.33, p < 0.001) and severe vision impairment (ARR = 3.59, 95% CI: 1.30–9.89, p = 0.01) and 10 times more likely to develop blindness (ARR = 9.65, 95% CI: 5.53–16.84, p < 0.001) after adjusting for age and gender (Table 5).

**Table 4:** Chi-squared test for the relationship between type of ocular trauma and visual status of patients.

Visual status	Type of Injury		Total N (%)
	Closed globe N (%)	Open globe N (%)	
Normal	161 (18.66)	183 (21.21)	344 (39.86)
Mild VI	29 (3.36)	90 (10.43)	119 (13.79)
Moderate VI	38 (4.40)	148 (17.15)	186 (21.55)
Severe VI	5 (0.58)	19 (2.20)	24 (2.78)
Blindness	16 (1.85)	174 (20.16)	190 (22.02)
Total	249 (28.85)	614 (71.15)	863 (100.00)

X^2^ = 100.98, df = 4, p < 0.001; VI: Vision impairment

**Table 5:** Multinomial logistic regression between type of ocular trauma and visual status of patients after adjusting for age and gender.

Variables		RRR (95% CI)	z-value	P-value
Normal (Base outcome)				
Mild VI	Open injury	2.915 (1.81–4.69)	4.41	**0.000**
	Age	1.016	2.60	0.000
	Gender	0.831	-0.78	0.434
Moderate VI	Open injury	3.550 (2.31–5.33)	5.88	**0.000**
	Age	1.004	0.72	0.473
	Gender	1.167	0.79	0.432
Severe VI	Open injury	3.588 (1.30–9.89)	2.47	**0.014**
	Age	1.014	1.22	0.224
	Gender	1.251	0.51	0.610
Blindness	Open injury	9.652 (5.53–16.84)	7.98	**0.000**
	Age	1.003	0.49	0.627
	Gender	0.907	-0.48	0.635

CI: Confidence interval, VI: Vision impairment

### Relationship between age groups and vision impairment

Whiles blindness was the most prevalent vision impairment due to ocular trauma among children (7.8%, 95% CI: 6.2%-9.8%) and the youth (8.0%, 95% CI: 6.4%-10.0%), mild (5.7%, 95% CI: 4.3%-7.4%) and moderate (5.7%, 95% CI: 4.3%-7.4%) vision impairment was most prevalent among adults (Table 6). For the elderly, moderate vision impairment (1.7%, 95% CI: 1.0%-2.9%) was found to be the most common vision impairment (Table 6). A chi-squared test revealed that there was also a statistically significant difference in vision impairment patterns among the different age groups (X^2^ = 36.19, df = 12, p < 0.001) (Table 7), while a multinomial regression analysis revealed the risk of developing vision impairment from ocular trauma based on age group. Ocular trauma occurring among the youth was 0.5 times more likely to result in moderate vision impairment (ARR = 0.49, 95% CI: 0.32–0.77, p = 0.002) and blindness (ARR = 0.53, 95% CI: 0.34–0.81, p = 0.004) than children after adjusting for gender and these were statistically significant. For adults, there was also a 0.5 times higher risk of ocular trauma resulting in blindness (ARR = 0.50, 95% CI: 0.31–0.82, p = 0.006) after adjusting for gender (Table 8), which was also statistically significant.

**Table 6:** Prevalence of presenting vision impairment among different age groups.

Presenting VI	Age groups			
	Children Prevalence (95% CI)	Youth Prevalence (95% CI)	Adult Prevalence (95% CI)	Elderly Prevalence (95% CI)
Normal	9.3 (7.5–11.4)	18.2 (15.8–20.9)	10.9 (9.0–13.2)	1.5 (0.9–2.6)
Mild VI	3.0 (2.1–4.4)	4.2 (3.0–5.7)	5.7 (4.3–7.4)	0.9 (0.4–1.9)
Moderate VI	7.2 (5.6–9.1)	7.0 (5.4–8.9)	5.7 (4.3–7.4)	1.7 (1.0–2.9)
Severe VI	0.7 (0.3–1.6)	1.0 (0.5–2.0)	0.7 (0.3–1.6)	0.4 (0.1–1.1)
Blindness	7.8 (6.2–9.8)	8.0 (6.4–10.0)	4.6 (3.4–6.3)	1.6 (0.8–3.2)

VI: Vision impairment, CI: Confidence interval

**Table 7:** Chi-squared test for the relationship between age groups and visual status of patients.

Visual Status	Age groups				Total N (%)
	Children N (%)	Youth N (%)	Adult N (%)	Elderly N (%)	
Normal	80 (9.27)	157 (18.19)	94 (10.89)	13 (1.51)	344 (39.86)
Mild VI	26 (3.01)	36 (4.17)	49 (5.68)	8 (0.93)	119 (13.79)
Moderate VI	62 (7.18)	60 (6.95)	49 (5.68)	15 (1.74)	186 (21.55)
Severe VI	6 (0.70)	9 (1.04)	6 (0.70)	3 (0.35)	24 (2.78)
Blindness	67 (7.76)	69 (8.00)	40 (4.63)	14 (1.62)	190 (22.02)
Total	241 (27.93)	331 (38.35)	238 (27.58)	53 (6.14)	863(100.00)

X^2^ = 36.19, df = 12, p < 0.001; VI: Vision impairment

**Table 8:** Multinomial logistic regression between age groups, ocular trauma and visual status of patients after adjusting for gender.

Variables		RRR (95% CI)	z-value	P-value
Normal (Base outcome)				
Mild VI	Youth	0.708	-1.18	0.237
	Adult	1.584	1.60	0.109
	Elderly	1.908	1.28	0.199
	Gender	0.826	-0.81	0.415
Moderate VI	Youth	0.492 (0.32–0.77)	-3.12	**0.002**
	Adult	0.677	-1.59	0.111
	Elderly	1.482	0.95	0.343
	Gender	1.108	0.53	0.594
Severe VI	Youth	0.762	-0.50	0.618
	Adult	0.859	-0.25	0.801
	Elderly	3.057	1.46	0.146
	Gender	1.162	0.34	0.733
Blindness	Youth	0.527 (0.34–0.81)	-2.92	**0.004**
	Adult	0.502 (0.31–0.82)	-2.74	**0.006**
	Elderly	1.296	0.62	0.537
	Gender	0.827	-0.96	0.335

CI: Confidence interval, VI: Vision impairment

## Discussion

The study found that there were more patients with open-globe injuries than closed-globe injuries in Zimbabwe. The prevalence of open-globe injuries among patients with ocular trauma from this hospital-based study was 71.2% and of this number, about 90% were caused by blunt trauma, while the rest were caused by penetrating, intraocular, and perforating injuries. This finding was similar to studies by Dogramaci, Erdur and Senturk [12], Nelson-Imoru et al. [13], and Mowatt et al. [14], which reported more cases of open-globe injuries (Table 9). Nonetheless, other studies have found that closed-globe injuries occur more frequently than open-globe injuries. For instance, in a Malaysian study by Mallika et al. [15], it was reported that closed-globe injuries accounted for 61.1% of all ocular trauma cases, whereas open-globe injuries made up 34.8%. Another study by Pandita et al. [16] in New Zealand also reported much more cases of closed-globe injury (568) than open-globe injury (253) (Table 9). A probable cause for this disparity might be attributed to the operational definition of ocular injuries used in the various studies.

**Table 9:** Comparative analysis of similar hospital-based studies on ocular trauma.

Authors	Country	Sample size	Sex (M/F)	Number of eyes	Age range	Number of open-globe injuries	Type of study
Current study	Zimbabwe	863	580/283	1007	0–87	614	Retrospective
Dogramaci et al [12]	Turkey	246	205/51	256	1–62	200	Retrospective
Nelson-Imoru and Mowatt [13]	Jamaica	80	57/23	84	3–64	Unclassified	Prospective
Mowatt et al [14]	Jamaica	252	198/54	252	17–92	126	Retrospective
Mallika et al [15]	Malaysia	233	200/33	257	1–72	Unclassified	Prospective
Pandita and Merriman [16]	New Zealand	821	608/213	821	0–98	253	Retrospective
Shah et al [17]	India	2607	1538/749	2607	0–92	451	Prospective

Notably, it was observed in this study that patients with open-globe injuries were approximately ten times more likely to develop blindness compared to those with closed-globe injuries, even after adjusting for age and gender (ARR = 9.65, 95% CI: 5.53–16.84, p < 0.001). This suggests that as more cases of open-globe injuries are prevented, more cases of blindness due to ocular trauma would be mitigated. As such, it is imperative that appropriate measures are put in place to prevent injuries from blunt trauma, penetrating, intraocular and perforating injuries. Also, the public must be educated on promptly reporting to the eye clinic whenever there is any form of trauma to the eye, as this could help to salvage vision appropriately. Several studies have reported that good visual acuity at the time of presentation in open-globe injuries results in good visual outcomes after treatment [18–24], and vice versa. Thus, it is important for clinicians to intensify public health education on the essence of prompt visit to the hospital by patients who experience any form of ocular trauma, particularly open-globe injuries as visual outcomes are highly dependent on visual acuity at the time of presentation.

The prevalence of vision impairment due to ocular trauma, on the other hand, was 60.1% which is quite remarkable given the detrimental effect (loss of livelihood, educational and occupational opportunities, quality of life, among others) vision impairment has on the individual and society at large. Blindness was the leading cause of vision impairment from ocular trauma, which suggested late presentation to the facility and the severity of the impact of trauma on the eye. Children and the youth also had the highest prevalence of blindness at presentation, which could be attributed to the nature of work among the youth and risky activities, for instance, playing with sharp objects, among others that children are most likely to be engaged. Another reason for this is that the youth are considered to be within the age group that is classified as active and most likely to overestimate their capabilities at their workplaces which, when ignored, could result in them experiencing several cases of ocular trauma. For instance, they are likely to be involved in the performance of more artisanal professions, uptick in assault cases, abuse of alcohol and drugs, and resistance to using safety equipment at work which are all potentially unsafe for their eyes. In Zimbabwe, agriculture and gold mining are the primary occupations of the residents [25] making accidents, including direct trauma with farm implements and other equipment, quite common. However, importantly, given the fact that the youth are the bedrock of every nation’s development, it is worth noting that eye health education as well as community and public sensitisation about ocular injuries, particularly in schools and workplaces, are promoted and intensified in order to ebb the prevalence of vision impairment that might occur due to ocular injuries.

The study also revealed that there were more males with ocular injuries than females in a ratio of 2:1, which was consistent with other similar studies [12, 13, 15, 17, 26–28] that reported a higher incidence of ocular injuries among males. For instance, while the study by Mallika et al. 015] reported a higher male-to-female ratio of 6:1, Dogramaci, Erdur, and Senturk [12] reported a ratio of 4:1. Nelson-Imoru et al. [13] and Shah et al. [17], however, reported comparable ratios (2.5:1 and 2.05:1, respectively) to this study. Considering that about two-thirds of males experienced ocular trauma of one kind or the other compared to females in this study, it could be extrapolated that males are more prone to ocular trauma than females, which is in line with findings by Stuart et al. [2] and Nelson-Imoru et al. [13]. Males are more likely to be involved in hazardous activities, hence, their higher proportion of ocular trauma cases than females.

Even though this study was limited by the fact that it was retrospective (secondary data), efforts were made to collect up-to-date and enough information to enhance appropriate and robust analyses. Another limitation of the study was that the classification of ocular trauma was not done based on confirmation from surgical findings, which would have been more accurate; however, preliminary diagnoses from the medical records were still sufficient to aid the categorisation of ocular trauma into different types. Visual acuity after treatment would have also been ideal in determining how specific types of ocular trauma affected visual outcomes of patients, however these were not available. Furthermore, there was no adequate information on closed-globe injuries as these types of injuries are less likely to report to a tertiary referral hospital. They would rather be treated in primary and secondary health facilities, thus missing data on them in the tertiary hospital. Other forms of closed injuries such as assaults from family and neighbours are also less likely to report to the hospital if they are presumed to be non-sight threatening. All these affected, which would have helped in the sub-classification of closed-globe injuries in this study. Despite the lack of these, the strength of the study was borne by the fact that all ocular trauma cases were adequately classified into open- and closed-globe injuries for analysis. Presenting distance visual acuities also enabled the categorisation of patients’ vision into the various forms of vision impairments due to ocular trauma, and the wide range of patients’ ages enabled the investigation of ocular trauma across a varying age group.

## Conclusion

There is a high prevalence of avoidable open-globe injuries in Zimbabwe with blunt trauma being the most significant cause. There is, therefore, the need to promote and intensify public eye health awareness and sensitisation on safety strategies for the prevention of ocular trauma throughout the country.

## Supporting information

S1 Checklist PLOS One STROBE checklist. (DOCX)

S1 Dataset Study data. (XLS)
